# Optimization of pineapple juice amount used as a negative oral contrast agent in magnetic resonance cholangiopancreatography

**DOI:** 10.1038/s41598-021-04609-6

**Published:** 2022-01-11

**Authors:** Matteo Renzulli, Daniele Caretti, Irene Pettinari, Maurizio Biselli, Stefano Brocchi, Alessandro Sergenti, Nicolò Brandi, Rita Golfieri

**Affiliations:** 1 Department of Radiology, IRCCS Azienda Ospedaliero-Universitaria di Bologna, Via Albertoni 15, Bologna, Italia; 2 “Toso Montanari” Industrial Chemistry Department, University of Bologna, Bologna, Italy; 3 Department of Medical and Surgical Sciences, Sant’Orsola Hospital, University of Bologna, Bologna, Italy; 4 Radiology Unit, Department of Diagnostic Medicine and Prevention, S. Orsola Hospital, University of Bologna, Bologna, Italy

**Keywords:** Gastroenterology, Chemistry

## Abstract

To evaluate the potential variability of Manganese (Mn^2+^) in commercial pineapple juice (PJ) produced in different years and to identify the optimal Mn^2+^ concentration in the correct amount of PJ to be administered prior to Magnetic Resonance Cholangiopancreatography (MRCP) in order to suppress the gastroduodenal (GD) liquid signal. The Mn^2+^ concentration in PJ produced in different years was defined using Atomic Absorption Spectrometry. The optimal Mn^2+^ concentration and the amount of PJ, were estimated in an in-vitro analysis, and were then prospectively tested in a population of patients who underwent MRCP. The results were compared with those achieved with the previous standard amount of PJ used in a similar population. The concentrations of Mn^2+^ in commercial PJ produced in different years did not differ. A total amount of 150 ml (one glass) of PJ having a high Mn^2+^ content (2.37 mg/dl) was sufficient for the suppression of the GD liquid signal, despite the additional dilution caused by GD liquids since it led to a final concentration of Mn^2+^ of 0.5–1.00 mg/dl. The optimized single-dose oral administration of 150 ml (approximately one glass) of PJ having a high Mn^2+^ concentration prior to MRCP was adequate to guarantee the correct amount of Mn^2+^ to suppress the GD signal.

## Introduction

Magnetic Resonance Cholangiopancreatography (MRCP) is the first-line non-invasive imaging technique for evaluating the biliary tract if immediate therapy for a known problem is not the primary aim^1^. Currently, MRCP has innumerable indications, such as evaluation of the normal biliary anatomy and its variants, and congenital and acquired biliary pathologies^2^.

Magnetic Resonance Cholangiopancreatography is performed with heavily T2-weighted (T2W) sequences, exploiting the fact that bile, as do all static fluids, has a long T2 relaxation time, making the bile ducts hyperintense and well recognisable (white) from the decreased signal of the background tissues (black), such as solid organs and flowing blood^3^.

A recognised limitation of MRCP is the possible overlap of the static fluids in the pancreatobiliary system and the static fluid in the gastrointestinal tract (i.e. the stomach, duodenum and proximal jejunum) which can obscure the distal portion of the common bile duct or simulate a disease^4,5^. Several oral negative contrast agents, containing paramagnetic substances which shorten the T2 relaxation time, thus reducing the signal hyperintensity of gastro-enteric fluids in standard MRCP sequences, have been produced by pharmaceutical companies and have been utilised in daily radiological practice in the past in order to overcome this limitation^6,7^. However, these negative oral contrast agents have many limitations; they are relatively unpalatable, are too diluted in the gastrointestinal tract or are too expensive^6^. To overcome these limitations, in more recent years, natural fruit juices have gained attention, the most popular being pineapple juice (PJ) and blueberry juice, owing to their properties which are similar to pharmaceutical oral negative contrast agents, having the same ability to suppress the high signal from gastrointestinal tract liquids on MRCP, but without the described limitations, having good palatability and a lower cost.

The ability of commercially available fruit juice to shorten the T2 relaxation time has recently been demonstrated to be closely dependent only on the Manganese (Mn^2+^) concentration and not on their Iron (III) concentration^8^. Moreover, it has also been demonstrated that all the commercial juice had a Mn^2+^ concentration capable of suppressing the gastrointestinal liquid signal on MRCP, and that PJ represents the optimal natural substance for suppressing gastrointestinal hyperintensity in heavily T2W sequences of MRCP due to its high Mn^2+^ content^8^.

However, to date, despite much effort to improve the image quality of MRCP, some questions still remain unanswered; it is unknown whether there is a variability in Mn^2+^ content in PJ of the same commercially available brand produced in different years. Furthermore, to date, the exact Mn^2+^ concentration and the juice dosage sufficient to suppress the gastroenteric liquid signal in fasting patients prior to MRCP has not been analysed or standardised to date.

This study has attempted to answer these three open issues. In particular, the primary aim of this study was to evaluate the potential variability of the Mn^2+^ content in PJ of the same commercially available brand produced in different years. The secondary aims were to identify and prospectively assess the optimal concentration of Mn^2+^ and the correct amount of PJ to be orally administered to fasting patients prior to MRCP in order to suppress the gastroduodenal (GD) liquid signal.

## Results

The concentrations of Mn^2+^ in PJ of the same commercially available brand produced in different years, redefined using AAS, did not demonstrate any difference. The absolute Mn^2+^ concentration measured was 2.37 mg/dl. This value did not differ with that measured in a previous study; in fact, the Mn^2+^ concentration in the PJ previously determined was 2.38 mg/dl^8^.

**Figure 1:**
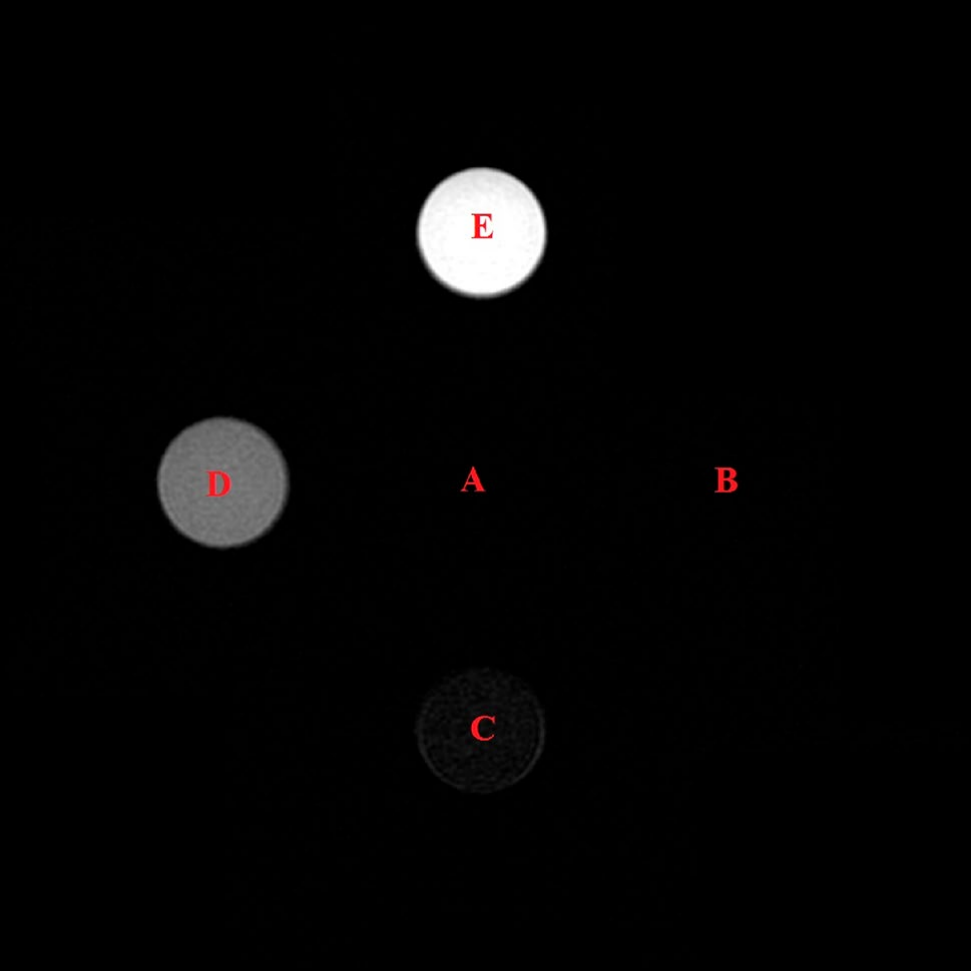
Magnetic resonance imaging evaluation (SSFSE TE: 1200 ms; TR 4000 ms) of five pure solutions with different Mn^2+^ concentrations.

In the in vitro analysis, the impact on the T2 values of the five pure chemical solutions containing decreasing Mn^2+^ concentrations (A–E) (from 1.00 to 0.063 mg/dl) is reported in Table 1. The analysis demonstrated that the solutions providing the best suppression of the GD liquid signal had a Mn^2+^ concentration ranging from 0.5 to 1.0 mg/dl (Table 1) (Fig. 1).

**Table 1:** Different dilutions of pure Mn^2+^ solutions and natural pineapple juice.

		Mn^2+^ concentration (mg/dl)	T2 value (ms)
Pure Mn^2+^ solution	A	1.00	87
	B	0.500	166
	C	0.250	314
	D	0.125	555
	E	0.063	849
Juice solution	F	1.00	95
	G	0.500	172
	H	0.250	330
	I	0.125	747
	J	0.063	1261

Moreover, the T2 values of the PJ solutions with the same dilutions of the pure Mn^2+^ solution are reported in Table 1. The impact on the T2 values of the five different solutions of PJ was practically the same as the five different chemical dilutions of the pure Mn^2+^ solutions and, therefore, the best suppression of the GD liquid signal was obtained with a Mn^2+^ concentration ranging from 0.5 to 1.0 mg/dl within the stomach and duodenum (Table 1).

For the clinical validation, the Authors relied on the results of the in vitro measurements: (a) 150 ml (1 full glass) of PJ contain 2.37 mg/dl of Mn^2+^ and (b) the best suppression of the GD liquid signal was obtained with a Mn^2+^ concentration ranging from 0.5 to 1.0 mg/dl. Assuming that the total GD fasting content does not exceed 200 ml, 150 ml of PJ (1 full glass) containing 2.37 mg/dl of Mn^2+^, when diluted in GD liquids, will reach an overall Mn^2+^ concentration of approximately 1 mg/dl.

The new selected dose of 1 glass of PJ was orally administered to n. 308 fasting patients (Group 1) who consecutively underwent MRCP. The results were compared to 305 patients (49.8% of the entire study population) which underwent MRCP after the oral intake of 300 ml of PJ (Group 2). The two groups were not different regarding gender (male 145/308, 47.1% vs. 145/305, 47.5%, respectively; *P* = 0.936) and age (66.5 years, IQR 19 years vs. 62 years, IQR 21.5 years, respectively; *P* = 0.059).

The indications for MRCP in the two groups are reported in Table 2. No statistical differences between the two groups were observed concerning the indications for MRCP which were a diagnosis of pancreatic Intraductal Papillary Mucinous Neoplasm and/or the follow-up of other biliopancreatic diseases (Group 1: 114/308, 37%; Group 2: 98/305, 32.1%) (Table 2).

**Table 2:** Indications for MRCP in the two groups of patients.

Indication	Group 1 glass (N = 308)	Group 2 glasses (N = 305)	*P*
Gallstones	83 (26.9%)	88 (28.9%)	0.653
Primary sclerosing cholangitis	48 (15.6%)	56 (18.4%)	0.390
Intraductal papillary mucinous neoplasm	114 (37%)	98 (32.1%)	0.234
Preoperative biliary anatomy evaluation	2 (0.6%)	8 (2.6%)	0.062
Liver transplantation	19 (6.2%)	22 (7.2%)	0.631
Others	42 (13.6%)	33 (10.8%)	0.325

**Figure 2:**
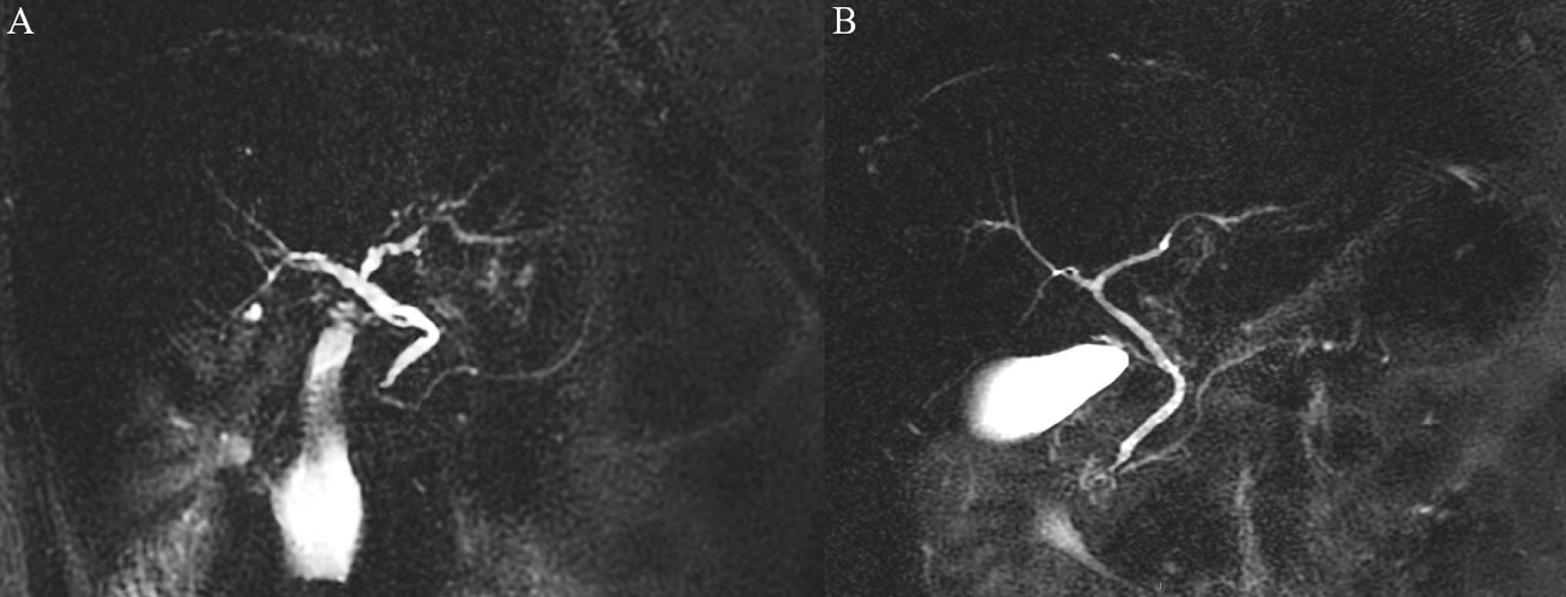
Magnetic Resonance Cholangiopancreatography of two different patients affected by the same pathology (Primary Sclerosing Cholangitis) performed after oral administration of one (**A**) or two glasses (**B**) of pineapple juice prior to the examination. No differences are evident between the two examinations in terms of suppression of the gastrointestinal liquid signal or in terms of imaging quality.

There were no statistical differences in the degree of suppression of the GD liquid signal between the two groups of patients after the analysis of the MRCP images (Fig. 2; Table 3). In particular, in Group 1, complete or good suppression of the GD liquid signal was observed in 294/308 patients (95.4%), not significantly different from Group 2 (Grade 1 plus Grade 2 observed in 280/305 patients, 91.8%; *P* = 0.07).

**Table 3:** Contrast effect based on the degree of suppression of the gastrointestinal liquid signal.

Degree of suppression of the gastrointestinal liquid signal	Group 1 (N = 308)	Group 2 (N = 305)
Complete	138 (44.8%)	138 (45.2%)
Good	156 (50.6%)	142 (46.6%)
Poor	13 (4.2%)	25 (8.2%)
Absent	1 (0.3%)	0

The evaluation of the Signal Intensity (SI) of the reference liquid (i.e. gallbladder, biliary tract, cerebrospinal fluid or renal cyst) (SIR) did not showed any difference between the two group of patients (group 1: SIR 2206, IQR 468 vs. group 2: SIR 2180, IQR 975; *P* = 0.455). No difference was observed regarding SI of GD fluid content in the stomach (SIS) and in the duodenum (SID) after oral administration of PJ between the two groups of patients (Table 4). Moreover, evaluation of the ratio between SIS and SID with SIR did not show any difference between the two groups (Table 4).

**Table 4:** Signal intensity values acquired after ingestion of the fruit juice.

Signal intensity (SI) values	Group 1 (N = 308)	Group 2 (N = 305)	*P*
SI stomach	76 (IQR 20)	72.5 (IQR 34)	0.069
SI duodenum	186 (IQR 41)	178.5 (IQR 108)	0.28
SI reference	2180 (IQR 975)	2206 (IQR 468)	0.455
SI ratio stomach/reference	3.31 (IQR 1.64)	3.08 (IQR 1.59)	0.057
SI ratio duodenum/reference	8.59 (IQR 4.04)	8.08 (IQR 5.79)	0.176

The overall quality of the MRCP images did not differ between the two groups as reported in Table 5. In particular, the Grade 1 quality (excellent image quality with high homogeneous signal intensity within the biliary lumen) was found in almost all the patients in both groups (Group 1: 282/308, 91.5%; Group 2: 291/305, 95.4%; *P* = 0.071).

**Table 5:** Quality of images acquired after ingestion of the fruit juice.

Quality of the images of the biliary lumen	Group 1 (N = 308)	Group 2 (N = 305)	*P*
Grade 1	282 (91.5%)	291 (95.4%)	0.071
Grade 2	20 (6.5%)	12 (3.9%)	0.203
Grade 3	3 (1%)	2 (0.7%)	1
Grade 4	3 (1%)	0	0.249

## Discussion

In recent years, considerable progress has been made in improving the quality of MRCP, allowing evaluation of both the normal biliary anatomy and its variants, and congenital and acquired benign and malignant biliary diseases using this imaging technique^2,9,10^. A recognised limitation of MRCP is represented by the possible overlap of the static liquids in the pancreatobiliary system and those in the gastrointestinal tract (i.e. the stomach, duodenum and proximal jejunum) which can obscure distal biliopancreatic ducts or simulate a disease having the same signal intensity on MRCP^4,5^.

Strong efforts have also been made to improve MRCP image quality and patient acceptance, such as the use of fruit juice as an oral negative contrast agent in order to overcome the limitations of oral chemical contrast media represented by their poor palatability and high cost. A recent paper has shown that Mn^2+^ alone represents the key element in fruit juices for suppressing the GD liquid signal and not Iron (III) as had previously been hypothesised^8^. Despite these advances, some open questions remain, In fact, it is unknown whether there is a variability in terms of the Mn^2+^ concentration in commercially available PJ of the same brand produced in different years. Furthermore, to date, the exact minimum concentration of Mn^2+^ and the correct amount of juice sufficient for obtaining signal suppression of the GD liquids after additional dilution in the stomach and in the duodenum in fasting patients prior to MRCP has not been analysed or standardised.

First of all, the present data showed the absence of variability in Mn^2+^ concentration in PJ of the same commercially available brand produced in different years. In the previous study, it had been demonstrated that there was no variability between PJ of different batches but of the same brand^8^. The combination of these two results is very important for standardisation regarding the adoption of PJ anywhere in the world since the concentration of Mn^2+^ will always be the same in the different batches of the same brand, regardless of the year of production of the PJ.

Secondly, not previously analysed in the scientific literature, this study started with an in-vitro analysis showing that the concentration of Mn^2+^ which permitted obtaining the best results in suppressing the GD liquid signal ranged from 0.5 to 1 mg/dl.

Lastly, the Authors considered the obvious dilution of ingested PJ in the GD content after fasting; the data related to the volume of liquids within the gastric lumen in a healthy subject after a 10-h fasting period were investigated, having a reported maximum volume of 96 ml^11^, the amount which should be added to a similar quantity of the fasting duodenal content. Therefore, the optimal amount of PJ to be administered orally before MRCP is that which allows obtaining a Mn^2+^ concentration of 0.5–1 mg/dl after additional dilution of the PJ caused by a mean total amount of 200 ml of GD liquids. The present study revealed that the oral administration, half an hour prior to MRCP, of only 1 glass (150 ml) of commercially available PJ, provided that it had a high Mn^2+^ content (2.37 mg/dl) was sufficient to obtain excellent suppression of the GD liquid signal without affecting the image quality of the examination. According to the best of the Authors’ knowledge, there are no previous studies which have analysed this topic. This amount was very important for the following reasons: (a) the amount of PJ administered orally is half of the dose previously recommended (2 glasses)^8^, thus halving the cost (b) the patient has to drink only one glass of juice which this can be of less discomfort in patients who find it difficult to drink for clinical reasons or because they do not like to drink pineapple juice due to its taste and (c) in the pandemic era, a single-patient/single-dose within a sealed Tetra Pak package is recommended in order to avoid contamination during the pouring of juice from a 1-l bottle of juice into a glass.

The clinical data of the present study were also important because they were tested on a large study population, including patients prospectively and consecutively enrolled in order to evaluate this new dosage for all possible clinical indications for MRCP. When comparing the two study groups, there were no statistically significant differences in terms of indications for the examination in the patients who received 1 glass of PJ prior to MRCP and those who received 2 glasses. Moreover, just as importantly, in more than 97% of cases, the assumption of PJ did not affect the overall quality of the MRCP. Finally, the oral assumption of only 1 glass of PJ can be recommended before performing MRCP for any indication without the risk of incurring a reduction in the image quality.

The main limitation of the present study was the non-prospective comparison between patients who assumed 1 glass of PJ (prospectively enrolled) and those who assumed 2 glasses of PJ (retrospectively collected). However, the necessary statistical tests were carried out to correctly identify any possible differences between the two groups without identifying any differences between the two populations in terms of demographic characteristics and indications for MRCP.

## Conclusion

There was no variability in the Mn^2+^ content of PJ of the same commercially available brand produced in different years. The Mn^2+^ concentration necessary to suppress the intensity of the GD liquid signal on MRCP ranged from 0.5 to 1.0 mg/dl. The oral administration of 150 ml (one glass) of PJ containing a high concentration of Mn^2+^ prior to MRCP is sufficient to guarantee the correct Mn^2+^ concentration to suppress the GD liquid signal, regardless of its additional dilution by gastrointestinal liquids.

## Methods

This study describes a standard of care change and was approved by the Institutional Review Board (Comitato Etico di Area Vasta Emilia Centro della Regione Emilia-Romagna) with registration number 791/2020/Oss/AOUBo (date of first registration: 11/05/2020). Written informed consent was obtained from all patients. This study was conducted according to the Declaration of Helsinki for clinical studies.

In vitro measurements were carried out to evaluate the potential variability of the Mn^2+^ content in PJ of the same commercially available brand produced in different years and to identify the optimal concentration of Mn^2+^ in the correct amount of PJ to be orally administered to fasting patients prior to MRCP in order to suppress the GD liquid signal.

Subsequent clinical validation was carried out to assess the secondary aim of the present study.

All the MRCP examinations were carried out according to the standardised protocol described in detail in a previously published paper^8^.

### In vitro measurements

The concentrations of Mn^2+^ in PJ of the same commercially available brand previously identified as having the highest Mn^2+^ content (2.38 mg/dl)^8^ were additionally redefined to exclude the possible variability of different periods of pineapple cultivation produced in different years utilising Atomic Absorption Spectrometry (AAS) with a Thermo iCE3300 AA spectrometer and acetylene/air flame atomisation.

A preliminary in vitro evaluation was carried out on a pure Mn^2+^ solution with different dilutions to identify the Mn^2+^ concentration sufficient to suppress the signal of intestinal liquids on MRCP. In particular, five solutions were prepared. A dose of 76.9 mg of Mn^2+^ sulphate monohydrate was weighed and dissolved in 500 ml of distilled water obtaining a solution which contained 5.00 mg/dl of Mn^2+^. Thereafter, 100 ml of this solution was diluted to 500 ml obtaining solution A containing 1.00 mg/dl of Mn^2+^. Additional dilutions were prepared to obtain solutions with decreasing concentrations of Mn^2+^: solution B with 0.50 mg/dl of Mn^2+^, solution C with 0.250 mg/dl of Mn^2+^, solution D with 0.125 mg/dl of Mn^2+^ and solution E with 0.063 mg/dl of Mn^2+^ (Table 1). Each solution was poured into small numbered cylinders, and the five cylinders containing the different concentrations of Mn^2+^ (Fig. 1) were inserted into the MRI scanner to measure the T2 values. The commonly used T2W sequences for MRCP were utilised; single shot fast-spin echo (SSFSE) T2W sequences (MRCP High Resolution Thick Slab) were carried out with an Echo Time (TE) value of 1200 ms and a Repetition Time (TR) of 4000 ms in order to calculate the T2 values of each of the different chemical solutions of Mn^2+^.

The concentration of Mn^2+^ in different commercial PJ had previously been determined^8^, and a known pineapple juice previously selected as having the highest Mn^2+^ content (2.38 mg/dl)^8^ was chosen for the in vivo study.

**Figure 3:**
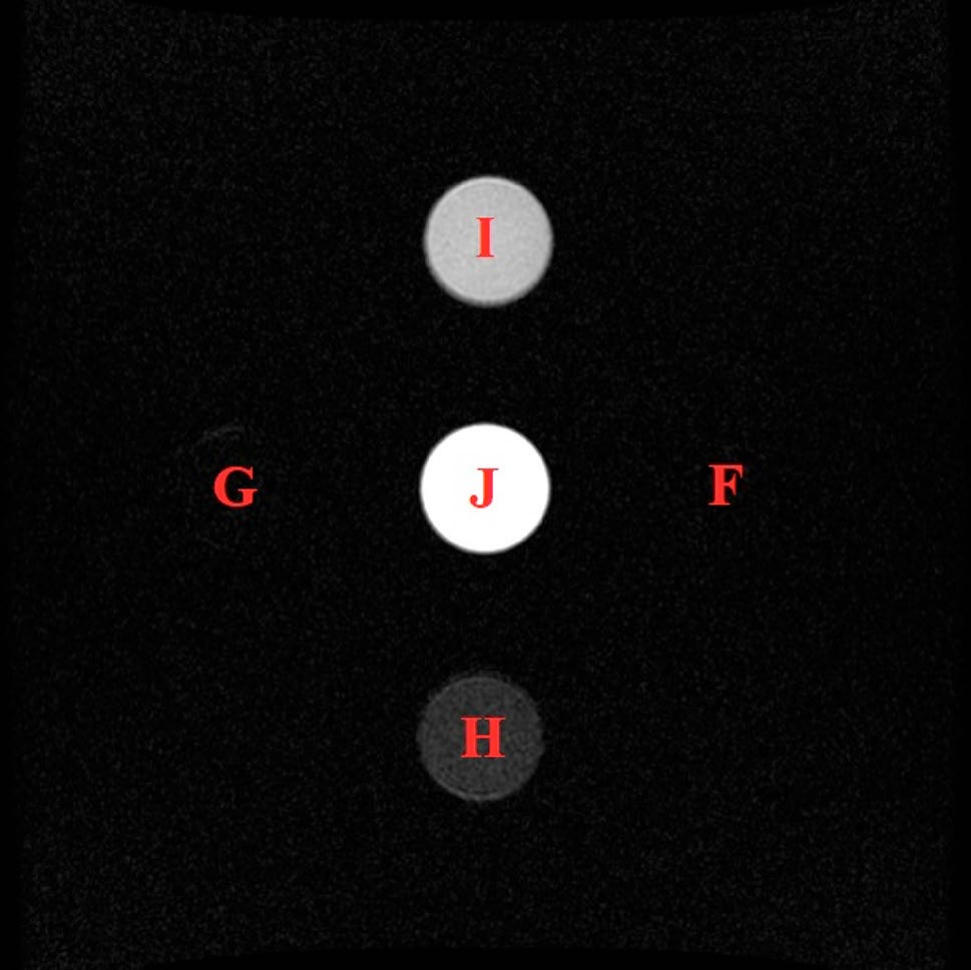
Magnetic resonance imaging evaluation (SSFSE TE: 1200 ms; TR 4000 ms) of five pineapple juices at different dilutions.

It was subsequently decided to analyse whether the ability to suppress the intestinal liquid signal on MRCP was the same between the pure Mn^2+^ solutions and the diluted PJ previously selected as having the highest Mn^2+^ content (2.38 mg/dl)^8^. Therefore, the PJ selected was diluted into different solutions to obtain the same Mn^2+^ concentration as the pure Mn^2+^ solutions. In particular, 85 ml of this PJ was diluted to 200 ml to obtain a Mn^2+^ concentration of 1 mg/dl (solution F). Additional dilutions permitted obtaining solutions G (0.5 mg/dl Mn^2+^), H (0.25 mg/dl Mn^2+^), I (0.125 mg/dl Mn^2+^) and J (0.063 mg/dl Mn^2+^). For these five solutions, the values of the T2W sequences were determined (Table 2) and imaged with the same MRCP sequence (Fig. 3).

### Clinical validation

A prospective clinical validation was carried out to assess the secondary aim of the study, i.e. the identification and prospective validation of the optimal Mn^2+^ concentration in the correct amount of PJ to be orally administered to fasting patients prior to MRCP in order to suppress the hyperintensity of the GD liquid.

Since the GD fluids diluted the orally administered PJ, the consolidated results concerning the residual fasting gastric content volume were analysed first. Considering that in healthy subjects who had a fasting period of at least 10 h, the gastric content volume ranged from 1 to 96 ml^11^, a gastric dilution of PJ into 100 ml of gastric fluids was prudentially assumed. It is well known that oral negative contrast agents are administered approximately half an hour before beginning MRCP. During this time interval, PJ could partially pass into the duodenum due to peristaltic movements and it could therefore be additionally diluted by the fasting duodenum content which has an estimated content similar to that of the stomach. Therefore, the total estimated fasting fluid content in the stomach plus duodenum was assumed to have a maximum value of 200 ml. Finally, according to these evaluations, it was decided to use a dose of PJ which contained a concentration of Mn^2+^ sufficient to suppress the GD signal also when additionally diluted in the GD fluids.

Subsequently, from December 2018 to June 2019, the new dose selected of PJ (derived from the in vitro analysis) was orally administered to fasting patients who consecutively underwent MRCP for any indication half an hour prior to the examination. The results were then compared with those of a previous population of patients who underwent MRCP between June 2018 and December 2018 in whom a standard dose of PJ of 300 ml (two glasses) was administered orally, as indicated in a previous study^8^. The sole exclusion criterion for enrolling patients in both groups was the presence of a biliodigestive anastomosis, i.e. patients in whom oral negative contrast agents are usually not administered prior to MRCP.

Two experienced radiologists (MR and SB) with more than ten years of experience in hepatobiliary and pancreatic imaging, blinded to the amount of PJ administered prior to MRCP, evaluated the results in a blinded fashion. A comparison between Group 1 (one glass) and Group 2 (two glasses), was carried out, based on the evaluation of the degree of reduction in the intensity of the GD liquid signal in the standard MRCP sequences. The suppression of the GD liquid signal was graded using a four-point scale: Grade 1 (complete suppression of the GD liquid signal); Grade 2 (good but not excellent suppression of the GD liquid signal); Grade 3 (very low suppression of the GD liquid signal) and Grade 4 (inadequate-absent suppression of the GD liquid signal).

The radiologists also evaluated the quality of all the MRCPs for each patient to avoid any possible impact of the PJ ingestion on the overall quality of the examination. The quality of the images, acquired after PJ ingestion, was graded using a four-point scale: Grade 1 (excellent image quality with high homogeneous signal intensity within the biliary lumen); Grade 2 (good but not excellent quality with good enhancement of the biliary lumen); Grade 3 (very low image quality with low enhancement of the biliary lumen) and Grade 4 (non-diagnostic quality with no or minimal signal intensity within the biliary lumen).

In an attempt to perform an image processing quantification of GD liquid signal suppression, the value of SI of a region of interest (ROI) of 0.5 cm^3^ was measured in 3 different sites: in the second portion of the duodenal lumen, in the region of the gastric fundus lumen and in correspondence of a reference liquid, meaning the location where the highest liquid intensity could be measured for each patient (such as gallbladder or biliary tract fluid, cerebro-spinal fluid or renal cyst content).

### Statistical analysis

The distribution asymmetry of the quantitative data was assessed using the Skewness test. The quantitative variables were expressed as mean ± standard deviation, or median and interquartile range (IQR), or ratio as appropriate. Comparisons between the groups were carried out using the χ^2^-test or the Fisher’s exact test (2-tailed) for the qualitative variables while the Mann–Whitney test was carried out for the quantitative variables. A *P* value of less than 0.05 was considered statistically significant. All the analyses were carried out using SPSS 23.0 software (IBM SPSS Statistics, USA).

### Ethical approval

Institutional Review Board (Comitato Etico di Area Vasta Emilia Centro della Regione Emilia-Romagna) approval was obtained with registration number 791/2020/Oss/AOUBo (date of first registration: 11/05/2020). This study was conducted according to the Declaration of Helsinki for clinical studies.

### Methodology

Prospective study describing a standard of care change, carried out at a single institute.

### Informed consent

Written informed consent was obtained from all patients.

## Data Availability

The datasets generated and analysed during the current study are available from the corresponding author on reasonable request.

## Abbreviations

MRCP: Magnetic Resonance Cholangiopancreatography
T2W: T2-weighted
PJ: Pineapple juice
Mn^2^^+^: Manganese
GD: Gastroduodenal
AAS: Atomic absorption spectrometry
SSFSE: Single shot fast-spin echo
TE: Echo time
TR: Repetition time
IQR: Interquartile range
